# Treatment of Streptozotocin-Induced Diabetic Rats with Alogliptin: Effect on Vascular and Neural Complications

**DOI:** 10.1155/2011/810469

**Published:** 2011-07-21

**Authors:** Eric P. Davidson, Lawrence J. Coppey, Brian Dake, Mark A. Yorek

**Affiliations:** ^1^Department of Internal Medicine, University of Iowa, Iowa City, IA 52246, USA; ^2^Department of Veterans Affairs Iowa City Health Care System, Iowa City, IA 52246, USA

## Abstract

We sought to determine the effect of dipeptidyl peptidase IV (DPP-IV) inhibition on streptozotocin diabetes-induced vascular and neural dysfunction. After 4 weeks of untreated diabetes, rats were treated for 12 weeks with Alogliptin (DPP-IV inhibitor). Diabetes caused a slowing of motor and sensory nerve conduction velocity, thermal hypoalgesia, reduction in intraepidermal nerve fiber density in the hindpaw, and impairment in vascular relaxation to acetylcholine and calcitonin gene-related peptide in epineurial arterioles. Treatment significantly improved motor nerve conduction velocity and thermal response latency. Sensory nerve conduction velocity was marginally improved with treatment of diabetic rats, and treatment did not improve the decrease in intraepidermal nerve fiber density. Vascular relaxation by epineurial arterioles to calcitonin gene-related peptide but not acetylcholine was significantly improved with treatment. These studies suggest that some but not all vascular and neural complications associated with type 1 diabetes can be improved with the inhibition of DPP-IV activity.

## 1. Introduction

My laboratory has focused on the role of microvascular dysfunction in the development and progression of diabetic neuropathy. Studies in types 1 and 2 diabetic rats have demonstrated that impaired vascular reactivity precedes the development of nerve dysfunction [1, 2]. In this study, we were interested in determining the potential benefits of treating type 1 diabetic rats with a dipeptidyl peptidase-IV inhibitor on microvascular and neural complications. 

Dipeptidyl peptidase-IV inhibitors alone or in combination with other therapies are being promoted for the treatment of type 2 diabetes, but little is known about potential benefits of these inhibitors in type 1 diabetes [3]. *In vivo* dipeptidyl peptidase-IV degrades and inactivates glucagon-like peptide-1 (GLP-1) and glucose-dependent insulinotropic polypeptide (GIP). These incretin hormones are released by gut endocrine cells and play an important role in glucose homeostasis primarily by regulating blood glucose levels through stimulating glucose-dependent insulin secretion, reducing glucagon secretion and slowing of gastric emptying [4, 5]. The degradation of these peptides by dipeptidyl peptidase-IV is responsible for their short half life in circulation [4, 6].

Dipeptidyl peptidase-IV or T-cell activation antigen CD26 is a serine exopeptidase that cleaves X-proline dipeptides from the N-terminus of polypeptides [7]. The enzyme is a type II transmembrane glycoprotein and is expressed on the surface of many cell types including the kidney, liver, and endothelial cells of blood vessels that drain the intestinal mucosa and can be found in soluble form in the circulation [6–9]. The inhibition of dipeptidyl peptidase-IV has been shown to improve glycemic control in patients with type 2 diabetes [8–10]. In rodent models of type 2 diabetes, inhibition of dipeptidyl peptidase-IV leads to improvement in glycemic control and is associated with an increase in glucose-dependent insulin secretion and restoration of *β* cell mass [10]. Dipeptidyl peptidase-IV inhibition of animal models of type 2 diabetes has also been associated with improving hyperlipidemia, inflammation, and hypertension [11]. For treatment of type 2 diabetes dipeptidyl peptidase-IV, inhibitors are becoming widely accepted because of their low risk of hypoglycemia; they are weight neutral, and appear to decrease *β*-cell apoptosis and increase *β*-cell survival. However, little information is available whether these inhibitors are effective in treatment of complications in animal models of type 1 diabetes [5]. In type 1 diabetes, insulin secretion is impaired due to loss of *β*-cells. Thus, any beneficial effects realized by dipeptidyl peptidase-IV inhibitors would be due to mechanisms other than increased insulin secretion. Using streptozotocin-treated diabetic rats, we sought to determine whether the inhibition of dipeptidyl peptidase-IV activity improves vascular or neural complications in type 1 diabetes. 

## 2. Materials and Methods

Unless stated otherwise, all chemicals used in these studies were obtained from Sigma Chemical Co. (St. Louis, MO).

### 2.1. Animals

Male Sprague-Dawley (Harlan Sprague Dawley, Indianapolis, ind, USA) rats 12 weeks of age were housed in a certified animal care facility, and food (Harlan Teklad, #7001, Madison, wis, USA) and water were provided ad libitum. All experiments were conducted in accordance with international standards on animal welfare and were compliant with all institutional and National Research Council's guidelines for use of animals (ACURF protocol 0210257). Diabetes was induced by intravenously injecting streptozotocin (55 mg/kg in 0.9% NaCl). Control rats were injected with vehicle alone. Diabetes was verified 72 h later by evaluating blood glucose levels with the use of glucose-oxidase reagent strips (Lifescan Inc., Milpitas, calif, USA). Rats having blood glucose level of 300 mg/dL (16.7 mM) or greater were considered to be diabetic.

Four weeks after the verification of diabetes two groups of diabetic, rats were established: an untreated group and a group treated with Alogliptin ((dipeptidyl peptidase-IV inhibitor) Takeda 200 mg/kg in the diet). The supplemental diet was prepared by mixing Alogliptin in the meal form of the diet followed by the formation of pellets and drying in a vacuum oven for 16 h at 37°C. The treatment phase of the study lasted 12 weeks. Alogliptin is a novel, orally active, quinazolinone-based, and noncovalent dipeptidyl peptidase-IV inhibitor. *In vitro* studies have indicated that Alogliptin exhibits more than 10,000 fold selectivity for dipeptidyl peptidase-IV over the closely related serine proteases dipeptidyl peptidase VIII or IX. This high selectivity may be important since the inhibition of dipeptidyl peptidase VIII or IX has been reported to be associated with multiorgan toxicity [4]. The pharmacokinetic, pharmacodynamic and efficacy profiles for Alogliptin has been reported in rats, dogs, and monkeys [4]. The dosage of Alogliptin used in this study was based on pharmacokinetic data [4]. Based on the amount of food consumed, diabetic rats treated with Alogliptin received about 10–20 mg/kg rat per day. For this study, all diabetic rats were treated with 2-3 units of Lantus insulin every other day in order to maintain weight. This dose of insulin did not correct hyperglycemia. Insulin treatment was discontinued 3 days prior to the terminal experiment. Serum insulin values were determined and were found to be very low or undetectable in both treated and untreated diabetic rats (data not shown). A set of control rats were also treated with Alogliptin, and results from these rats demonstrated that Alogliptin had no effect on neural or vascular function of epineurial arterioles (data not shown).

### 2.2. Thermal Nociceptive Response

On the day before terminal studies, thermal nociceptive response in the hindpaw was measured using the Hargreaves method as previously described [12].

### 2.3. Motor and Sensory Nerve Conduction Velocity and Biological and Oxidative Stress Markers. 

On the day of terminal studies, rats were weighed and anesthetized with Nembutal i.p. (50 mg/kg, i.p., Abbott Laboratories, North Chicago, ill, USA). Nonfasting blood glucose was determined. Hemoglobin A_1_C levels were determined using a Glyco-tek affinity column kit (Helena Laboratories, Beaumont, tex, USA). Serum samples were collected for determination, of free fatty acid, triglyceride, and free cholesterol, using commercial kits from Roche Diagnostics, Mannheim, Germany; Sigma Chemical Co., St. Louis, mlo, USA; and Bio Vision, Mountain View, calif, USA, respectively. Serum thiobarbituric acid reactive substances (TBARS) levels were determined as a marker of oxidative stress as previously described [13]. Serum dipeptidyl peptidase-IV activity was measured using a kit from eBioscience (Vienna, Austria). This assay was performed to confirm that Alogliptin decreased dipeptidyl peptidase-IV activity in treated rats. Data in Table 2 demonstrates that diabetes caused an increase in dipeptidyl peptidase-IV activity in serum, but this was not significant. Treating diabetic rats with Alogliptin caused a significant decrease in serum dipeptidyl peptidase-IV activity.

Motor nerve conduction velocity (MNCV) was determined as previously described using a noninvasive procedure in the sciatic-posterior tibial conducting system [1]. Sensory nerve conduction velocity (SNCV) was determined using the digital nerve to the second toe as described by Obrosova et al. [14]. The MNCV and SNCV were reported in meters per second.

### 2.4. Intraepidermal Nerve Fiber Density in the Hindpaw

Immunoreactive intraepidermal nerve fiber profiles were visualized using confocal microscopy. Samples of skin of the right hindpaw were fixed, dehydrated, and embedded in paraffin. Sections (7 *μ*m) were collected and immuno stained with anti-PGP9.5 antibody (rabbit antihuman, AbD serotic, Morpho Sys US Inc., Raleigh, nc, USA) overnight followed by treatment with secondary antibody Alexa Fluor 546 goat anti rabbit (Invitrogen, Eugene, ore, USA). Profiles were counted by two individual investigators that were blinded to the sample identity. All immunoreactive profiles within the epidermis were counted and normalized to epidermal length [15, 16].

### 2.5. Vascular Reactivity in Epineurial Arterioles

Videomicroscopy was used to investigate *in vitro* vasodilatory responsiveness of epineurial arterioles vascularizing the region of the sciatic nerve as previously described [16–19]. Cumulative concentration-response relationships were evaluated for acetylcholine (10^−8^–10^−4^ M) and calcitonin gene, related peptide (10^−11^–10^−8^ M) using vessels from each group of rats. At the end of each dose response curve for acetylcholine or calcitonin gene, related peptide, papaverine (10^−5^ M) was added to determine maximal vasodilation.

### 2.6. Data Analysis

Results are presented as mean ± SEM. Comparisons between the groups were conducted using one-way ANOVA and Bonferroni after test (Prism software; GraphPad, San Diego, calif, USA). Concentration response curves for acetylcholine and calcitonin gene-related peptide were compared using a two-way repeated measure analysis of variance with autoregressive covariance structure using proc mixed program of SAS [17, 18]. A *P* value of less than 0.05 was considered significant.

## 3. Results

### 3.1. Effect of Treatment of Streptozotocin-Diabetic Rats with Alogliptin on Weight and Blood Glucose

Data in Table 1 demonstrate that untreated or diabetic rats treated with Alogliptin had reduced weight gain and at the end of the study period weighed significantly less than control rats. All diabetic rats were hyperglycemic at the end of the study period as indicated by significantly elevated blood glucose and hemoglobin A_1_C levels (Table 1).

### 3.2. Effect of Treatment of Streptozotocin-Diabetic Rats with Alogliptin on Serum Lipid and TBARS Levels and ACE Activity

Data in Table 2 demonstrate that serum TBARS, a marker for oxidative stress, was significantly increased in diabetic rats. Treating diabetic rats with Alogliptin lowered serum TBARS, but the difference was not significant compared to untreated diabetic rats. Diabetes caused a significant increase in serum triglycerides, free fatty acids, and cholesterol levels. Treating diabetic rats with Alogliptin did not significantly reduce the hyperlipidemia.

### 3.3. Effect of Treatment of Streptozotocin-Diabetic Rats with Alogliptin on Nerve Conduction Velocity, Thermal Nociception, and Intraepidermal Nerve Fiber Density

**Figure 1:**
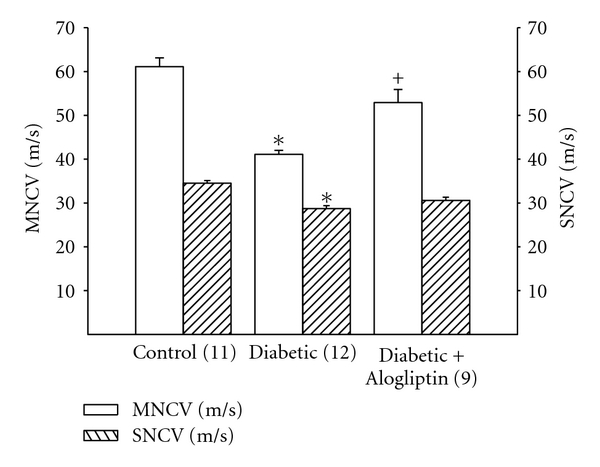
Effect of treatment of streptozotocin-diabetic rats with Alogliptin on motor and sensory nerve conduction velocity. At 12 weeks of age rats were made diabetic using streptozotocin and 4 weeks later were divided into 2 groups. One group was fed a normal diet and the other group was fed a diet containing Alogliptin for 12 weeks. A nondiabetic control group was also included. Afterwards, motor and sensory nerve conduction velocity was examined as described. Data are presented as the mean ± SEM in m/sec. The number of rats in each group was the same as shown in Table 1. *P < 0.05, compared to rats fed the standard diet (control), ^+^ *P* < 0.05, compared to untreated diabetic rats.

**Figure 2:**
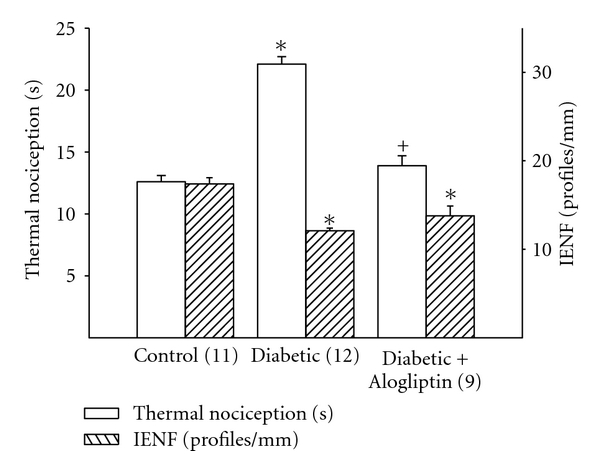
Effect of treatment of streptozotocin-diabetic rats with Alogliptin on thermal nociception and intraepidermal nerve fiber density. Rats were treated as describe in Figure 1. Thermal nociception and intraepidermal nerve fiber density was examined as described. Data are presented as the mean ± SEM for thermal nociception in sec and intraepidermal nerve fiber profiles per mm. The number of rats in each group was the same as shown in Table 1. *P < 0.05, compared to rats fed the standard diet (control), ^+^ *P* < 0.05, compared to untreated diabetic rats.

Motor and sensory nerve conduction velocity was significantly decreased in diabetic rats (Figure 1). Treating diabetic rats with Alogliptin was more effective in improving motor nerve conduction velocity than sensory nerve conduction velocity. Data in Figure 2 demonstrate that after 16 weeks of diabetes rats are hypoalgesic and treating diabetic rats with Alogliptin improved thermal nociception. After 16 weeks of untreated diabetes, there was a decrease in intraepidermal nerve fiber profiles in the hindpaw (Figure 2). Treating diabetic rats with Alogliptin did not significantly improve innervation of the hindpaw.

### 3.4. Effect of Treatment of Streptozotocin-Diabetic Rats with Alogliptin on Vascular Relaxation by Epineurial Arterioles

**Figure 3:**
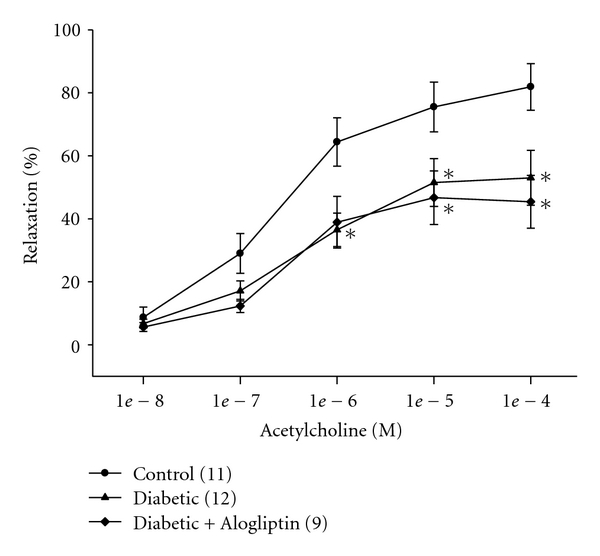
Effect of treatment of streptozotocin-diabetic rats with Alogliptin on vascular relaxation by acetylcholine in epineurial arterioles. Rats were treated as describe in Figure 1. Pressurized arterioles (40 mm Hg and ranging from 60 to 100 *μ*m luminal diameter) were constricted with U46619 (30–50%), and incremental doses of acetylcholine were added to the bathing solution while recording steady state vessel diameter. Data are presented as the mean of % relaxation ± SEM. For these studies, two vessels were collected from each rat, studied, and the data combined. The number of rats in each group was the same as shown in Table 1. *P < 0.05, compared to rats fed the standard diet (control).

In these studies, treatment with Alogliptin did not prevent impairment of acetylcholine-mediated vascular relaxation by epineurial arterioles from diabetic rats (Figure 3). 

**Figure 4:**
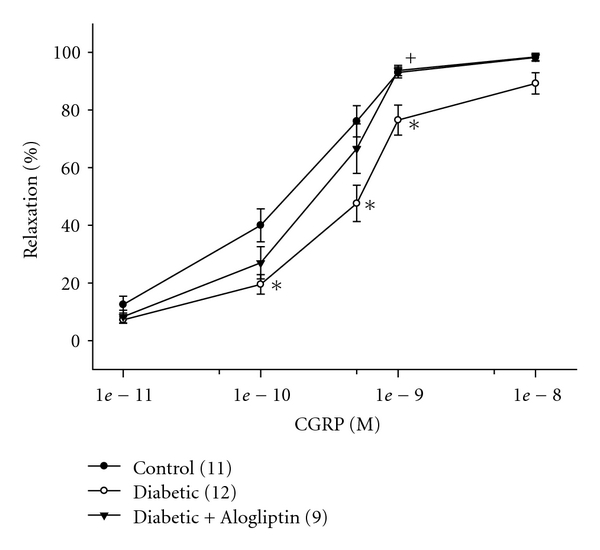
Effect of treatment of streptozotocin-diabetic rats with Alogliptin on vascular relaxation by calcitonin gene-related peptide in epineurial arterioles*. *Arterioles were derived from rats as described in Figure 3. Incremental doses of calcitonin gene-related peptide (CGRP) were added to the bathing solution while recording steady state vessel diameter. Data are presented as the mean of % relaxation ± SEM. The number of rats in each group was the same as shown in Table 1. *P < 0.05, compared to rats fed the standard diet (control), ^+^ *P* < 0.05, compared to untreated diabetic rats.

Figure 4 provides data on the effect of diabetes and treatment with Alogliptin on vascular relaxation mediated by calcitonin gene-related peptide in epineurial arterioles. Calcitonin gene-related peptide is the most potent vasodilator of epineurial arterioles that we have identified, and the potency is decreased by chronic diabetes [19]. Treatment of diabetic rats with Alogliptin significantly improved calcitonin gene-related peptide-mediated vascular relaxation in epineurial arterioles (Figure 4).

## 4. Discussion

We previously reported that the inhibition of neutral endopeptidase was an efficacious treatment for vascular and neural complications in streptozotocin diabetic rats [20, 21]. We attributed the efficacy of the inhibition of neutral endopeptidase to the protection of vasoactive peptides from degradation [20–22]. We have also previously shown that the inhibition of angiotensin converting enzyme was beneficial in treating vascular and neural dysfunction in streptozotocin diabetic rats [18]. Both neutral endopeptidase -and angiotensin- converting enzyme are proteases whose expression is increased in diabetes [23]. 

Dipeptidyl peptidase is another protease whose activity may impact on the progression of diabetic complications. inhibition of dipeptidyl peptidase IV has been promoted as a treatment for type 2 diabetes [24–27]. In ob/ob mice, chronic dipeptidyl peptidase IV inhibition increased GLP-1 levels and improved *β*-cell function and glycemic control and decreased triglycerides [28, 29]. In the high fat fed/streptozotocin ICR (imprinting control region) mouse, a nongenetic model of type 2 diabetes, the inhibition of dipeptidyl peptidase IV improved glycemic control and increased *β*-cell function [10]. In the streptozotocin-nicotinamide diabetic mouse or rat, rodent models for type 2 diabetes, dipeptidyl peptidase IV inhibition improved hemoglobin A_1_C levels, glucose intolerance, and lipid profiles and increased GLP-1 levels [30, 31]. In Zucker diabetic fatty rats, a genetic rodent model for type 2 diabetes, the inhibition of dipeptidyl peptidase IV corrected glycemic dysmetabolism, hypertriglyceridemia, inflammation, and hypertension [11]. Thus, it is well documented that dipeptidyl peptidase IV inhibition is an effective treatment for type 2 diabetes although little is known about the effect of chronic dipeptidyl peptidase IV inhibitor treatment on animal models of type 1 diabetes. 

As we have previously reported that streptozotocin-induced type 1 diabetes was associated with decreased motor and sensory nerve conduction velocity, hypoalgesia, decrease in intraepidermal nerve fiber density and decreased vascular relaxation in response to acetylcholine and calcitonin gene-related peptide by epineurial arterioles [20, 21]. The present studies demonstrated that treatment with Alogliptin for 12 weeks, following 4 weeks of untreated diabetes, improved motor and to a lesser extent sensory nerve conduction velocity and thermal hypoalgesia but not nerve fiber density. Previously, we have shown that pretreating rats or mice with 3-O-methylglucose (5.5 mmol/kg body weight) immediately prior to the injection of streptozotocin prevented hyperglycemia and development of neural complications [32, 33]. Thus, it is unlikely that the onset of diabetes-related complications in streptozotocin-treated rodents is due to streptozotocin toxicity. These rats also had very low to nondetectable serum insulin levels that were not improved with Alogliptin (data not shown). Thus, the beneficial effects of Alogliptin treatment were likely due to changes unrelated to improved insulin release and action.

Chronic treatment of streptozotocin-diabetic rats with Alogliptin improved vascular function in epineurial arterioles in response to calcitonin gene-related peptide but not to acetylcholine in epineurial arterioles. The inability of Alogliptin treatment to improve acetylcholine-mediated vascular relaxation is likely due to the lack of effect by Alogliptin to reduce oxidative stress. It has been shown that dipeptidyl peptidase IV converts brain-derived natriuretic peptide into its short form which lacks vasodilatory activity [26]. It is possible that Alogliptin may prevent degradation of natriuretic peptides and perhaps calcitonin gene-related peptide thereby preserving their vasodilatory properties. Dipeptidyl peptidase IV also degrades other peptides with vascular function, and preserving their activity in diabetic rats could also improve vascular function and reduce ischemia. In this regard, we found that Alogliptin treatment improved motor and to a lesser extent sensory nerve conduction velocity and thermal nociception but did not improve intraepidermal nerve fiber density. Moreover, unlike many rodent models of type 2 diabetes, treatment of type 1 diabetic rats with a dipeptidyl peptidase inhibitor did not improve hyperlipidemia or glycemic control. We attribute the improvement in motor nerve conduction velocity by Alogliptin to improved vascular relaxation in response to calcitonin gene-related peptide.

Our results indicate an apparent dissociation between improvement in thermal hypoalgesia and intraepidermal nerve fiber density. Even though, we and others have shown that drug treatment of diabetic rodents can improve both sensory perception and intraepidermal nerve fiber density, the current studies using treatment with Alogliptin did not support this finding [20, 21, 34–36]. A similar result was obtained in streptozotocin-diabetic C57Bl6/J mice treated with Baicalein [37]. Beiswenger et al. [38] in a study using streptozotocin-diabetic C57Bl6/J mice reported that thermal hypoalgesia developed before a measureable reduction of intraepidermal nerve fiber density. Pain perception involves a complex signaling transduction pathway involving neuropeptides such as calcitonin gene-related peptide and substance P. In these studies, we have demonstrated that treatment with Alogliptin protected vascular relaxation in response in calcitonin gene-related peptide in epineurial arterioles. It is possible that Alogliptin also protected the bioactivity and signaling cascade mediated by calcitonin gene-related peptide and perhaps substance P involved in pain perception. The reduction of intraepidermal nerve fibers in these studies was about 30%. Thus, sufficient fibers may remain to initiate normal pain perception as long as the signaling cascade is not compromised.

Ours is not the first study to examine the effect of dipeptidyl peptidase IV inhibitor treatment of type 1 diabetic rats. Jin et al. [39] reported that dipeptidyl peptidase IV inhibitor treatment of type 1 diabetic rats for 32 weeks prevented peripheral nerve degeneration. They concluded that preserving GLP-1 levels may be useful in peripheral neuropathy. Alogliptin treatment of diabetic rats in our study reduced dipeptidyl peptidase IV activity, but we did not measure the activity of GLP-1. Like the studies by Jin et al. [39], our studies also demonstrated that treatment of type 1 diabetic rats with a dipeptidyl peptidase IV inhibitor partially preserved neural function. Some of our results also defer, for instance intraepidermal nerve fiber loss was not prevented in our studies [39]. This could be due to some important differences in the two study designs. Jin et al. initiated their studies using much younger animals [39]. Pathophysiology of diabetes complications may differ depending on the age of animals at the onset of diabetes. In both studies treatment was initiated four weeks after the induction of diabetes, and our dose of inhibitor and the high dose used by Jin et al. were similar although the duration of treatments was different. Our treatment period lasted 12 weeks, whereas the treatment period in the study by Jin et al. was 32 weeks [39]. It could be that a longer treatment period would provide more time for nerve regeneration. The drug used and delivery were also different in the two studies. In our study, Alogliptin was delivered in the diet, and in Jin et al. vildagliptin was given once daily in water [39]. These differences may account for why we did not observe the protection of intraepidermal nerve fiber density in our studies. 

## 5. Conclusions

Studies were performed of the effect of treating streptozotocin type 1 diabetic rats with Alogliptin, a dipeptidyl peptidase IV inhibitor, on vascular and nerve dysfunction. We found that treatment with Alogliptin improved some neural and vascular complications. It is becoming clear that dipeptidyl peptidase IV inhibitors have multiple affects and may improve outcome by mechanisms unrelated to the preservation of GLP-1 or GIP [40]. 

##  Conflict of Interests

The authors have no conflict of interests to report.

## Figures and Tables

**Table 1:** Effect of treatment of streptozotocin-diabetic rats with alogliptin on change in body weight, blood glucose and hemoglobin A_1_C.

Determination	Control (11)	Diabetic (12)	Diabetic + Alogliptin (9)
Start weight (g)	359 ± 4	362 ± 4	358 ± 6
End weight (g)	524 ± 9	334 ± 14^a^	373 ± 30^a^
Blood glucose (mg/dL)	110 ± 5	578 ± 13^a^	536 ± 23^a^
Hb A_1_C (%)	5.3 ± 0.3	13.9 ± 0.4^a^	13.1 ± 1.4^a^

Data are presented as the mean ± SEM. ^a^ *P* < 0.05 compared to control. Parentheses indicate the number of experimental animals.

**Table 2:** Effect of treatment of streptozotocin-diabetic rats with alogliptin on serum thio barbituric acid reactive substances, triglycerides, free fatty acids, cholesterol and dipeptidyl peptidase-IV activity.

Determination	Control (11)	Diabetic (12)	Diabetic + Alogliptin (9)
TBARS (*μ*g/mL)	0.78 ± 0.07	1.07 ± 0.06^a^	0.93 ± 0.12
Triglycerides (mg/dL)	25 ± 3	206 ± 27^a^	225 ± 46^a^
Free fatty acids (mmol/L)	0.11 ± 0.03	0.56 ± 0.07^a^	0.41 ± 0.11^a^
Cholesterol (mg/dL)	82 ± 13	223 ± 38^a^	175 ± 35^a^
Dipeptidyl peptidase-IV activity (ng/mL)	24.0 ± 3.0	35.4 ± 5.1	18.3 ± 3.4^b^

Data are presented as the mean ± SEM. ^a^ *P* < 0.05 compared to control; ^b^ *P* < 0.05 compared to diabetic. Parentheses indicate the number of experimental animals.

## References

[1] Coppey LJ, Davidson EP, Dunlap JA, Lund DD, Yorek MA (2000). Slowing of motor nerve conduction velocity in streptozotocin-induced diabetic rats is preceded by impaired vasodilation in arterioles that overlie the sciatic nerve. *Experimental Diabesity Research*.

[2] Coppey LJ, Gellett JS, Davidson EP, Dunlap JA, Yorek MA (2002). Effect of treating streptozotocin-induced diabetic rats with sorbinil, myo-inositol or aminoguanidine on endoneurial blood flow, motor nerve conduction velocity and vascular function of epineurial arterioles of the sciatis nerve. *International Journal of Experimental Diabetes Research*.

[3] Davidson JA (2010). Incorporating incretin-based therapies into clinical practice: differences between glucagon-like peptide 1 receptor agonists and dipeptidyl peptidase 4 inhibitors. *Mayo Clinic Proceedings*.

[4] Lee B, Shi L, Kassel DB, Asakawa T, Takeuchi K, Christopher RJ (2008). Pharmacokinetic, pharmacodynamic, and efficacy profiles of alogliptin, a novel inhibitor of dipeptidyl peptidase-4, in rats, dogs, and monkeys. *European Journal of Pharmacology*.

[5] Neumiller JJ, Wood L, Campbell RK (2010). Dipeptidyl peptidase-4 inhibitors for the treatment of type 2 diabetes mellitus. *Pharmacotherapy*.

[6] Lam S, Saad M (2010). Saxagliptin: a new dipeptidyl peptidase-4 inhibitor for type 2 diabetes. *Cardiology in Review*.

[7] Matteucci E, Giampietro O (2009). Dipeptidyl peptidase-4 (CD26): knowing the function before inhibiting the enzyme. *Current Medicinal Chemistry*.

[8] Deacon CF, Toft-Nielsen M, Pridal L, Willms B, Holst JJ (1995). Both subcutaneously and intravenously administered glucagon-like peptide I are rapidly degraded from the NH2-terminus in type II diabetic patients and in healthy subjects. *Diabetes*.

[9] Kieffer TJ, McIntosh CHS, Pederson RA (1995). Degradation of glucose-dependent insulinotropic polypeptide and truncated glucagon-like peptide 1 in vitro and in vivo by dipeptidyl peptidase IV. *Endocrinology*.

[10] Mu J, Petrov A, Eiermann GJ (2009). Inhibition of DPP-4 with sitagliptin improves glycemic control and restores islet cell mass and function in a rodent model of type 2 diabetes. *European Journal of Pharmacology*.

[11] Ferreira L, Teixeira-De-Lemos E, Pinto F (2010). Effects of sitagliptin treatment on dysmetabolism, inflammation, and oxidative stress in an animal model of type 2 diabetes (ZDF rat). *Mediators of Inflammation*.

[12] Oltman CL, Davidson EP, Coppey LJ, Kleinschmidt TL, Lund DD, Yorek MA (2008). Attenuation of vascular/neural dysfunction in zucker rats treated with enalapril or rosuvastatin. *Obesity*.

[13] Oltman CL, Coppey LJ, Gellett JS, Davidson EP, Lund DD, Yorek MA (2005). Progression of vascular and neural dysfunction in sciatic nerves of Zucker diabetic fatty and Zucker rats. *American Journal of Physiology*.

[14] Obrosova IG, Li F, Abatan OI (2004). Role of poly(ADP-ribose) polymerase activation in diabetic neuropathy. *Diabetes*.

[15] Beiswenger KK, Calcutt NA, Mizisin AP (2008). Epidermal nerve fiber quantification in the assessment of diabetic neuropathy. *Acta Histochemica*.

[16] Davidson EP, Coppey LJ, Calcutt NA, Oltman CL, Yorek MA (2010). Diet-induced obesity in Sprague-Dawley rats causes microvascular and neural dysfunction. *Diabetes/Metabolism Research and Reviews*.

[17] Coppey LJ, Gellett JS, Davidson EP, Dunlap JA, Lund DD, Yorek MA (2001). Effect of antioxidant treatment of streptozotocin-induced diabetic rats on endoneurial blood flow, motor nerve conduction velocity, and vascular reactivity of epineurial arterioles of the sciatic nerve. *Diabetes*.

[18] Coppey LJ, Davidson EP, Rinehart TW (2006). ACE inhibitor or angiotensin II receptor antagonist attenuates diabetic neuropathy in streptozotocin-induced diabetic rats. *Diabetes*.

[19] Yorek MA, Coppey LJ, Gellett JS, Davidson EP (2004). Sensory nerve innervation of epineurial arterioles of the sciatic nerve containing calcitonin gene-related peptide: effect of streptozotocin-induced diabetes. *Experimental Diabesity Research*.

[20] Davidson EP, Kleinschmidt TL, Oltman CL, Lund DD, Yorek MA (2007). Treatment of streptozotocin-induced diabetic rats with AVE7688, a vasopeptidase inhibitor: effect on vascular and neural disease. *Diabetes*.

[21] Oltman CL, Davidson EP, Coppey LJ, Kleinschmidt TL, Dake B, Yorek MA (2011). Role of the effect of inhibition of neutral endopeptidase on vascular and neural complications in streptozotocin-induced diabetic rats. *European Journal of Pharmacology*.

[22] Davidson EP, Coppey LJ, Kleinschmidt TL, Oltman CL, Yorek MA (2009). Vascular and neural dysfunctions in obese Zucker rats: effect of AVE7688. *Experimental Diabetes Research*.

[23] Yorek MA (2008). The potential role of angiotensin converting enzyme and vasopeptidase inhibitors in the treatment of diabetic neuropathy. *Current Drug Targets*.

[24] Doupis J, Veves A (2008). DPP4 inhibitors: a new approach in diabetes treatment.. *Advances in Therapy*.

[25] Ahrén B (2008). Emerging dipeptidyl peptidase-4 inhibitors for the treatment of diabetes. *Expert Opinion on Emerging Drugs*.

[26] Lambeir AM, Scharpé S, De Meester I (2008). DPP4 inhibitors for diabetes-What next?. *Biochemical Pharmacology*.

[27] Verspohl EJ (2009). Novel therapeutics for type 2 diabetes: incretin hormone mimetics (glucagon-like peptide-1 receptor agonists) and dipeptidyl peptidase-4 inhibitors. *Pharmacology and Therapeutics*.

[28] Moritoh Y, Takeuchi K, Asakawa T, Kataoka O, Odaka H (2008). Chronic administration of alogliptin, a novel, potent, and highly selective dipeptidyl peptidase-4 inhibitor, improves glycemic control and beta-cell function in obese diabetic ob/ob mice. *European Journal of Pharmacology*.

[29] Moritoh Y, Takeuchi K, Hazama M (2009). Chronic administration of voglibose, an *α*-glucosidase inhibitor, increases active glucagon-like peptide-1 levels by increasing its secretion and decreasing dipeptidyl peptidase-4 activity in ob/ob mice. *Journal of Pharmacology and Experimental Therapeutics*.

[30] Tahara A, Matsuyama-Yokono A, Nakano R, Someya Y, Hayakawa M, Shibasaki M (2009). Antihyperglycemic effects of ASP8497 in streptozotocin-nicotinamide induced diabetic rats: comparison with other dipeptidyl peptidase-IV inhibitors. *Pharmacological Reports*.

[31] Matsuyama-Yokono A, Tahara A, Nakano R, Someya Y, Hayakawa M, Shibasaki M (2009). Chronic inhibition of dipeptidyl peptidase-IV with ASP8497 improved the HbA1c level, glucose intolerance, and lipid parameter level in streptozotocin-nicotinamide-induced diabetic mice. *Naunyn-Schmiedeberg’s Archives of Pharmacology*.

[32] Wiese TJ, Lowe WL, Yorek MA (1996). Regulation of the Na+/myo-inositol cotransporter mRNA levels in streptozotocin-induced diabetic rats and by hyperglycemia in cultured cells. *International Journal of Diabetes*.

[33] Davidson E, Coppey L, Lu B (2009). The roles of streptozotocin neurotoxicity and neutral endopeptidase in murine experimental diabetic neuropathy. *Experimental Diabetes Research*.

[34] Sugimoto K, Kojima K, Baba M, Yasujima M (2011). Olmesartan ameliorates peripheral nerve dysfunction in Zucker diabetic fatty rats. *Journal of Hypertension*.

[35] Obrosova IG, Xu W, Lyzogubov VV (2008). PARP inhibition or gene deficiency counteracts intraepidermal nerve fiber loss and neuropathic pain in advanced diabetic neuropathy. *Free Radical Biology and Medicine*.

[36] Drel VR, Pacher P, Vareniuk I (2007). A peroxynitrite decomposition catalyst counteracts sensory neuropathy in streptozotocin-diabetic mice. *European Journal of Pharmacology*.

[37] Stavniichuk R, Drel VR, Shevalye H (2011). Baicalein alleviates diabetic peripheral neuropathy through inhibition of oxidative-nitrosative stress and p38 MAPK activation. *Experimental Neurology*.

[38] Beiswenger KK, Calcutt NA, Mizisin AP (2008). Dissociation of thermal hypoalgesia and epidermal denervation in streptozotocin-diabetic mice. *Neuroscience Letters*.

[39] Jin HY, Liu WJ, Park JH, Baek HS, Park TS (2009). Effect of Dipeptidyl Peptidase-IV (DPP-IV) Inhibitor (Vildagliptin) on Peripheral Nerves in Streptozotocin-induced Diabetic Rats. *Archives of Medical Research*.

[40] Nauck MA, El-Ouaghlidi A (2005). The therapeutic actions of DPP-IV inhibition are not mediated by glucagon-like peptide-1. *Diabetologia*.

